# Supplementary material for: Virion morphology and on-virus spike protein structures of diverse SARS-CoV-2 variants
Source: EMBO J. 2024 Nov 14;43(24):6469–95. doi: 10.1038/s44318-024-00303-1 (PMC11649927; doi:10.1038/s44318-024-00303-1)
Supplement: Supplementary file 1 — Appendix [file 44318_2024_303_MOESM1_ESM.pdf]

Appendix: Figures and Table

Virion morphology and on-virus spike protein structures of diverse SARS-CoV-2 variants

Zunlong Ke[1,2,3], Thomas P. Peacock[4,5], Jonathan C. Brown[4], Carol M. Sheppard[4], Tristan I. Croll[6,7], Abhay Kotecha[8], Daniel H. Goldhill[4,9], Wendy S. Barclay[4], John A.G. Briggs[1,2,*]

[1] Department of Cell and Virus Structure, Max Planck Institute of Biochemistry, Martinsried, Germany

[2] Structural Studies Division, Medical Research Council Laboratory of Molecular Biology, Cambridge, UK

[3] Department of Molecular Biosciences, The University of Texas at Austin, Austin, TX, USA.

[4] Department of Infectious Disease, Imperial College London, London, UK

[5] The Pirbright Institute, Woking, UK

[6] Cambridge Institute for Medical Research, University of Cambridge, Cambridge, UK

[7] Altos Labs, Cambridge, UK

[8] Materials and Structural Analysis, Thermo Fisher Scientific, Eindhoven, the Netherlands

[9] Department of Pathobiology and Population Sciences, Royal Veterinary College, London, UK.

* Correspondence to JAGB: briggs@biochem.mpg.de

Contents:

**Appendix Figure S1.**	Workflows for cryo-EM structure determination.	2-7
**Appendix Figure S2.**	Examples of density maps fitted with PDB models.	8
**Appendix Figure S3.**	Examples of density maps fitted with PDB models.	9
**Appendix Figure S4.**	FSC curves for reported structures.	10
**Appendix Figure S5.**	Euler angular distribution for the particles contributing to the structures of the five variants.	11
**Appendix Table S1.**	Cryo-EM data collection, image processing, and refinement statistics.	12

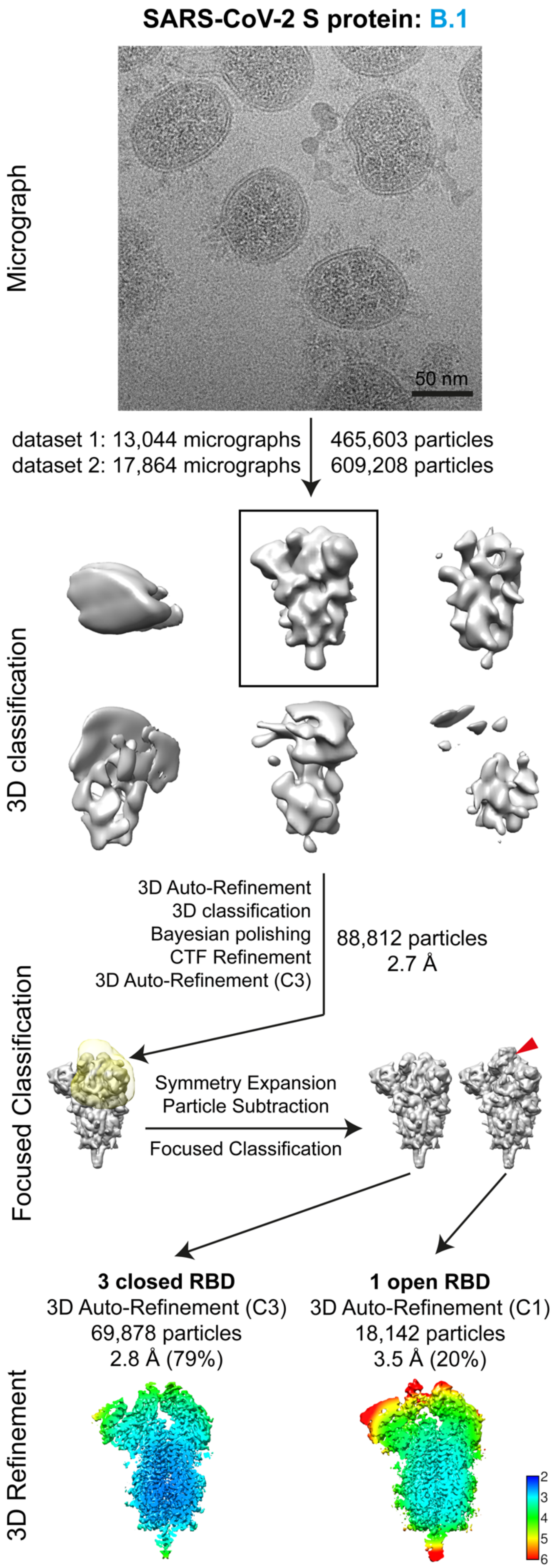
SARS-CoV-2 S protein: B.1
Micrograph
50 nm
dataset 1: 13,044 micrographs
dataset 2: 17,864 micrographs
465,603 particles
609,208 particles
3D classification
3D Auto-Refinement
3D classification
Bayesian polishing
CTF Refinement
3D Auto-Refinement (C3)
88,812 particles
2.7 Å
Focused Classification
Symmetry Expansion
Particle Subtraction
Focused Classification
3 closed RBD
3D Auto-Refinement (C3)
69,878 particles
2.8 Å (79%)
1 open RBD
3D Auto-Refinement (C1)
18,142 particles
3.5 Å (20%)
3D Refinement
2
3
4
5
6

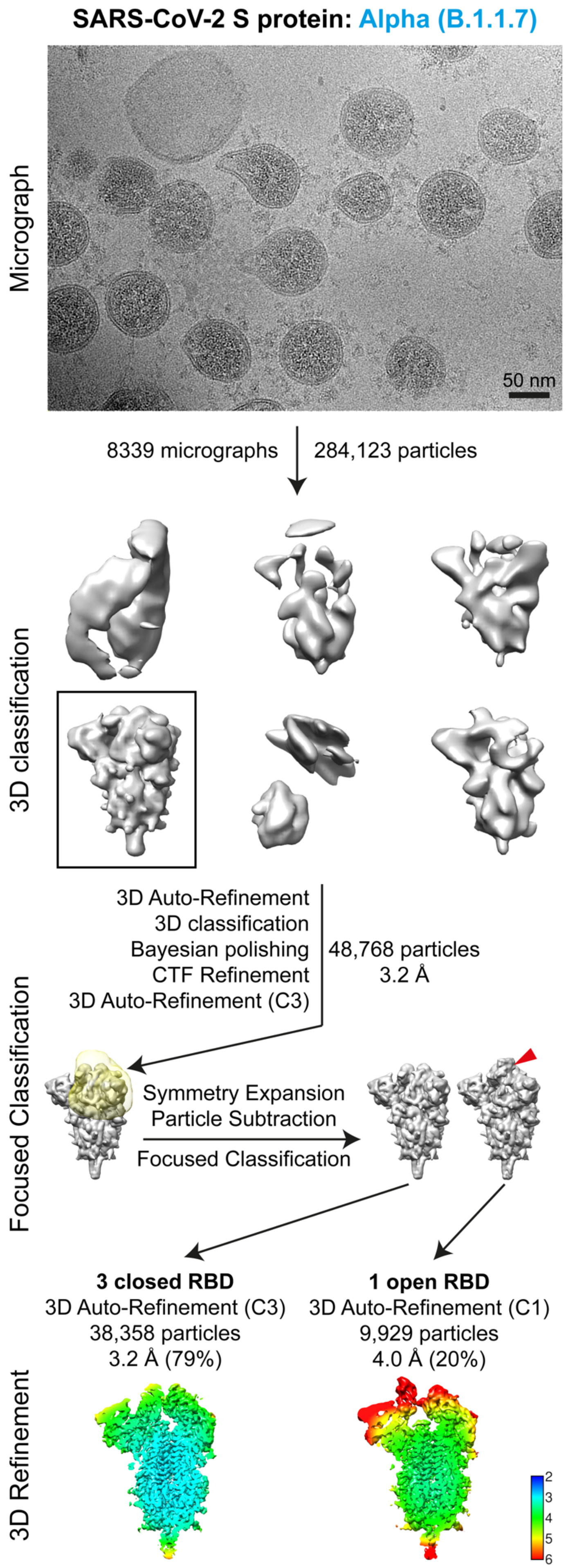

SARS-CoV-2 S protein: Alpha (B.1.1.7)
Micrograph
50 nm
8339 micrographs
284,123 particles
3D classification
3D Auto-Refinement
3D classification
Bayesian polishing
CTF Refinement
3D Auto-Refinement (C3)
48,768 particles
3.2 Å
Focused Classification
Symmetry Expansion
Particle Subtraction
Focused Classification
3 closed RBD
3D Auto-Refinement (C3)
38,358 particles
3.2 Å (79%)
1 open RBD
3D Auto-Refinement (C1)
9,929 particles
4.0 Å (20%)
3D Refinement
2
3
4
5
6

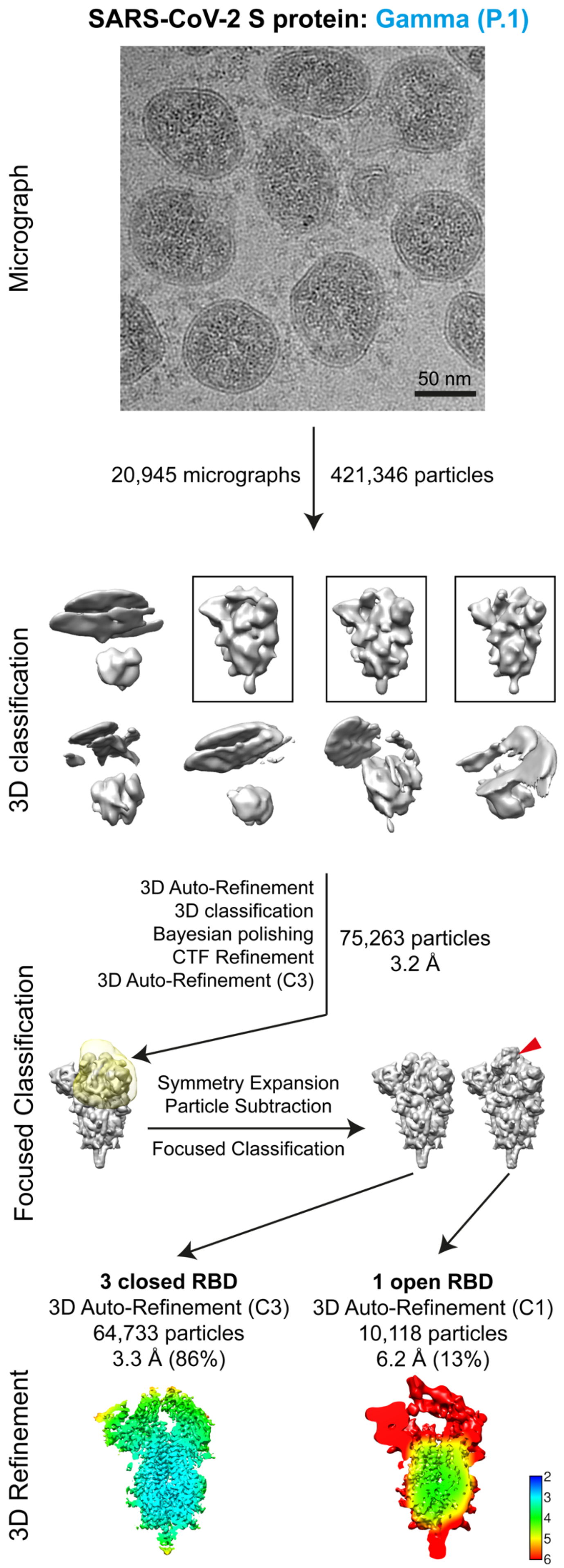
SARS-CoV-2 S protein: Gamma (P.1)
Micrograph
50 nm
20,945 micrographs
421,346 particles
3D classification
3D Auto-Refinement
3D classification
Bayesian polishing
CTF Refinement
3D Auto-Refinement (C3)
75,263 particles
3.2 Å
Focused Classification
Symmetry Expansion
Particle Subtraction
Focused Classification
3 closed RBD
3D Auto-Refinement (C3)
64,733 particles
3.3 Å (86%)
1 open RBD
3D Auto-Refinement (C1)
10,118 particles
6.2 Å (13%)
3D Refinement
2
3
4
5
6

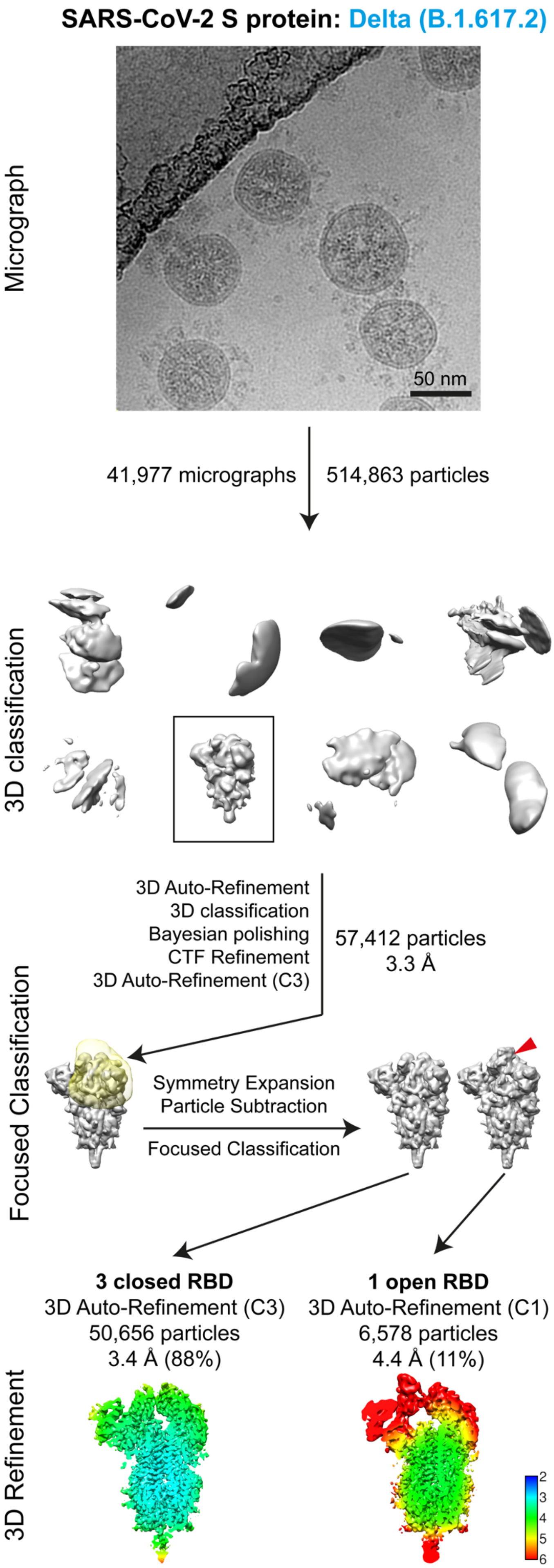
SARS-CoV-2 S protein: Delta (B.1.617.2)
Micrograph
50 nm
41,977 micrographs
514,863 particles
3D classification
3D Auto-Refinement
3D classification
Bayesian polishing
CTF Refinement
3D Auto-Refinement (C3)
57,412 particles
3.3 Å
Focused Classification
Symmetry Expansion
Particle Subtraction
Focused Classification
3 closed RBD
3D Auto-Refinement (C3)
50,656 particles
3.4 Å (88%)
1 open RBD
3D Auto-Refinement (C1)
6,578 particles
4.4 Å (11%)
3D Refinement
2
3
4
5
6

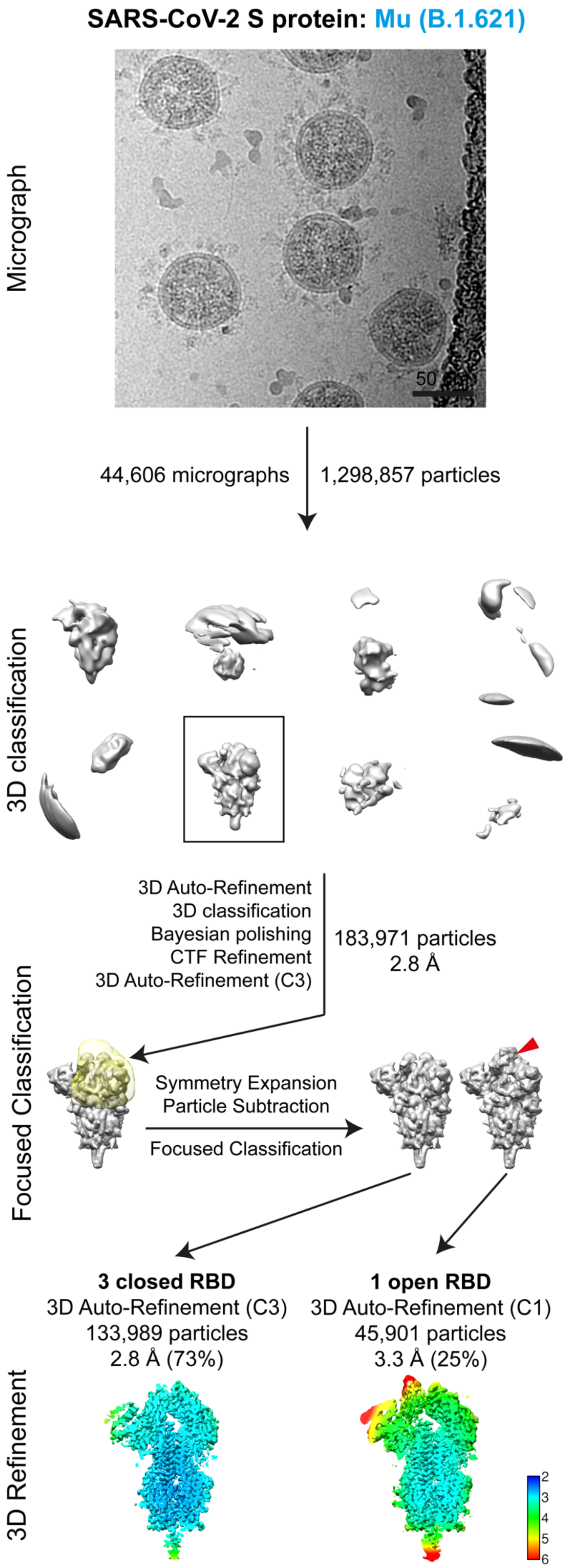
SARS-CoV-2 S protein: Mu (B.1.621)
Micrograph
50 nm
44,606 micrographs
1,298,857 particles
3D classification
3D Auto-Refinement
3D classification
Bayesian polishing
CTF Refinement
3D Auto-Refinement (C3)
183,971 particles
2.8 Å
Focused Classification
Symmetry Expansion
Particle Subtraction
Focused Classification
3 closed RBD
3D Auto-Refinement (C3)
133,989 particles
2.8 Å (73%)
1 open RBD
3D Auto-Refinement (C1)
45,901 particles
3.3 Å (25%)
3D Refinement
2
3
4
5
6

Appendix Figure S1. Workflows for cryo-EM structure determination. An overview of the major image processing steps involved and the number of particles considered at each stage, including 3D classification, focused 3D classification, and 3D auto-refinement. The percentage next to the resolution in each local resolution map indicates the ratio of the corresponding S trimer conformations. Half-map FSC curves and model-map FSC curves are illustrated in **Appendix Figure S4**.

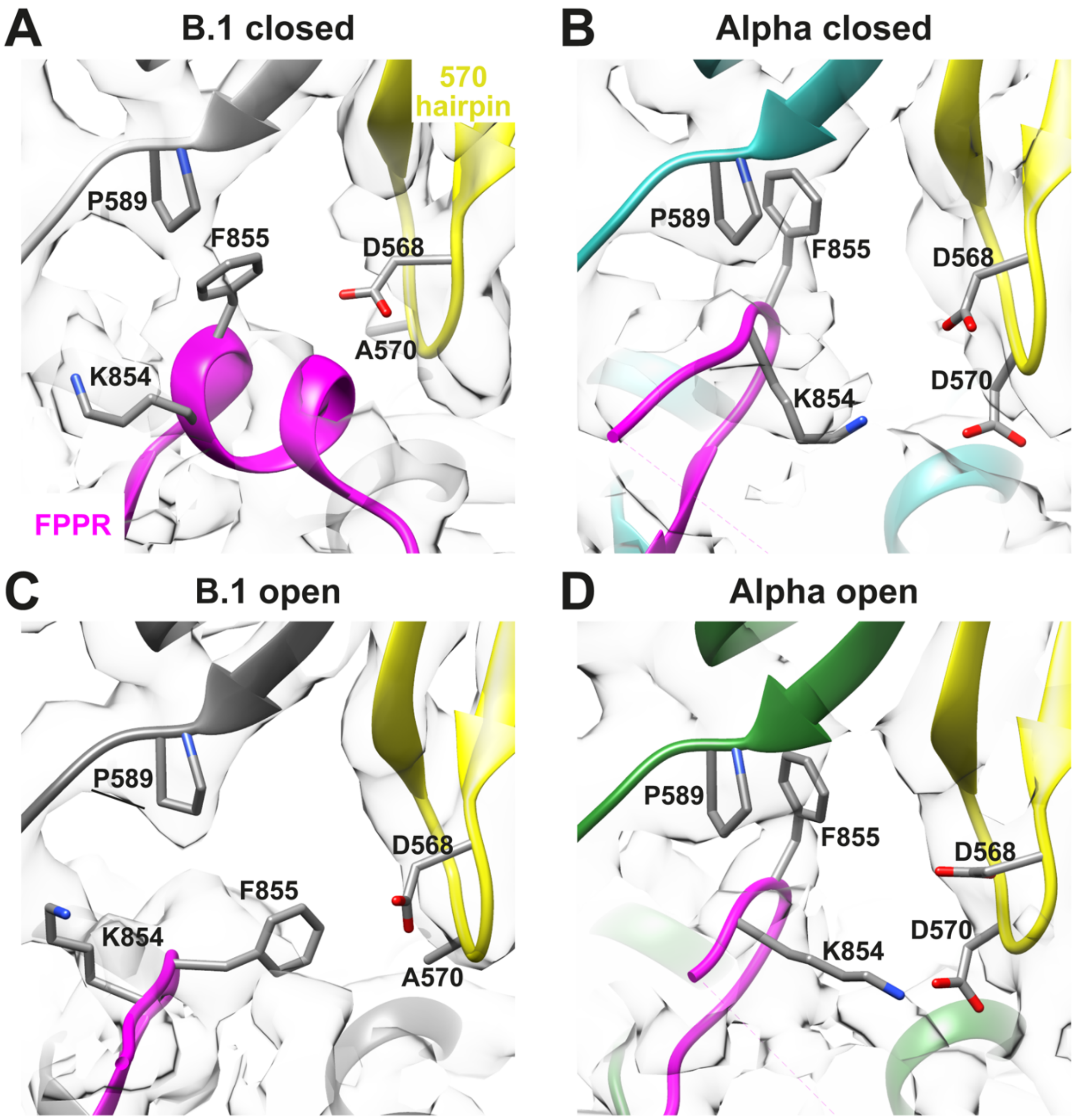

Appendix Figure S2. Examples of density maps fitted with PDB models. Zoom-in views of **Figure 5** to illustrate the key structural motifs and residues in the A570D mutation region where 570 hairpin and FPPR motif are color coded in yellow and magenta, respectively. The key residues Pro 589, Phe 855, Lys 854, Asp 568, and Ala 570 (B.1 variant) or Asp 570 (Alpha variant) are illustrated for each map.

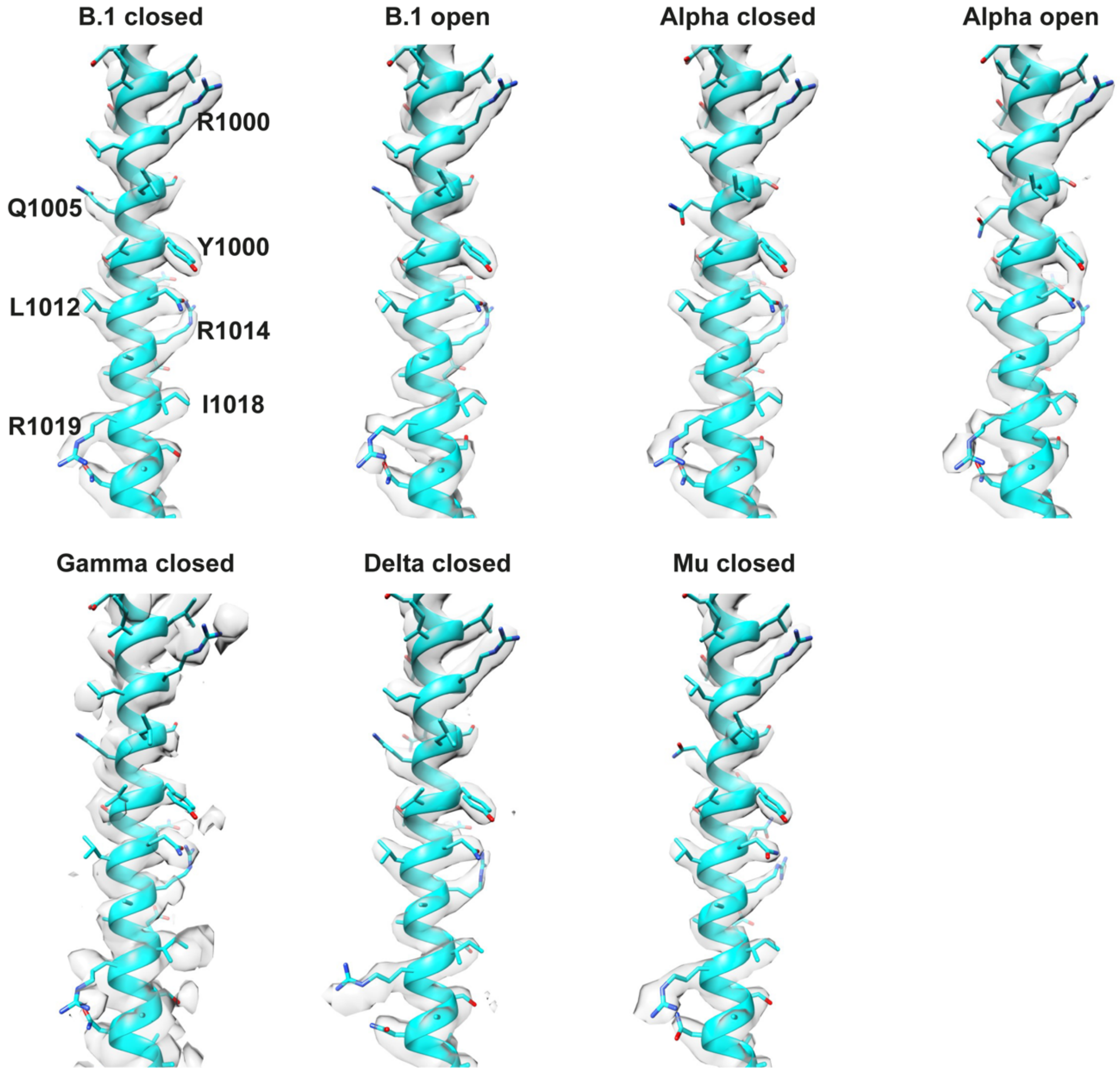

Appendix Figure S3. Examples of density maps fitted with PDB models. The central helix density is shown, fitted with the molecular model, for each of the seven maps discussed in the manuscript. The same region is shown for all maps. Some amino acid side chains are labeled in B.1 closed density map.

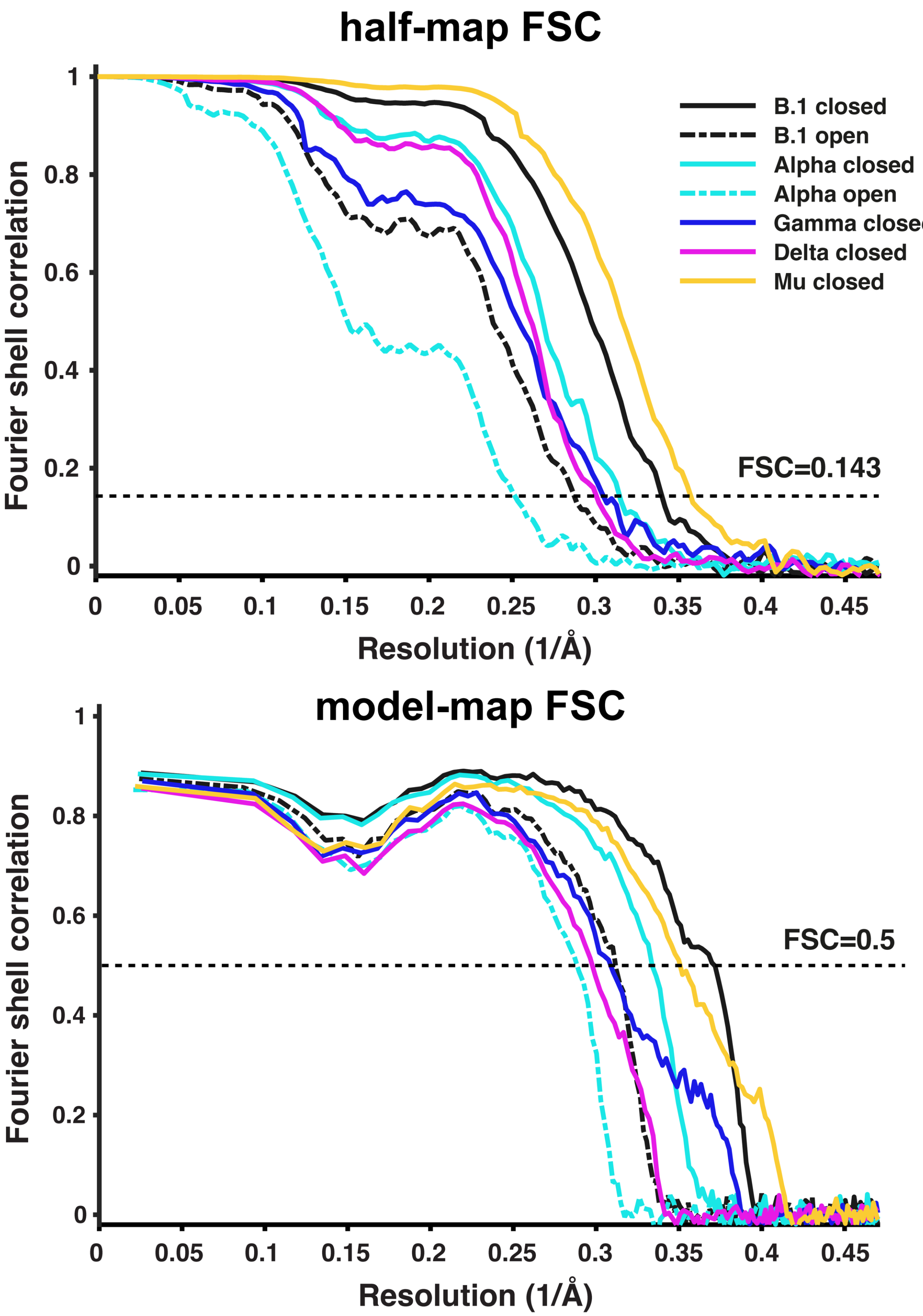

Appendix Figure S4. FSC curves for reported structures. Half-map FSC (top panel, FSC=0.143) and model-map FSC (bottom panel, FSC=0.5) for the seven reported structures. The top and bottom panels share the same color schemes. Note, because the 1-open RBD models (B.1 and Alpha) were built based on the higher-resolution 3-RBD closed structures, the model-map correlation for B.1 open and Alpha open is higher than expected from the half-map FSC. See also methods section and **Appendix Table S1**.

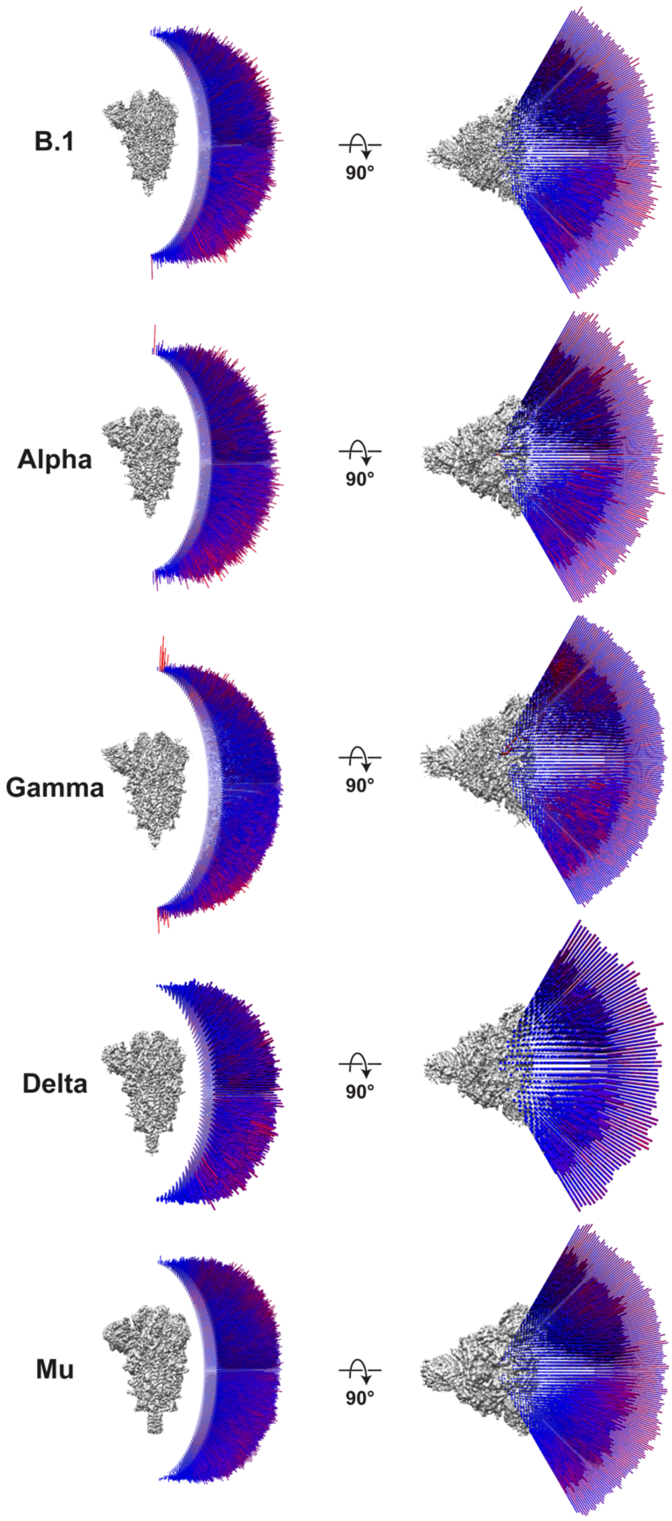

Appendix Figure S5. Euler angular distribution for the particles contributing to the structures of the five variants. The angular distribution is from C3 symmetry applied structures. The Euler angle distribution is not uniform because particles are all from the edges of virions and therefore views along the symmetry axis are lacking. These views are not required to obtain isotropic reconstructions and Fourier space is properly filled in this scenario. The output is from the Auto Refinement job from RELION.

Appendix Table S1. Cryo-EM data collection, image processing, and refinement statistics.

	B.1		Alpha		Gamma	Delta	Mu
**RBD conformation**	**closed EMD-45863 PDB 9CRC**	**open EMD-45864 PDB 9CRD**	**closed EMD-45865 PDB 9CRE**	**open EMD-45866 PDB 9CRF**	**closed EMD-45867 PDB 9CRG**	**closed EMD-45868 PDB 9CRH**	**closed EMD-45869 PDB 9CRI**
**Data collection and processing**							
Microscope	Titan Krios G3		Titan Krios G3		Titan Krios G4	Titan Krios G3i	Titan Krios G3i
Microscope location	MRC-LMB Cambridge		MRC-LMB Cambridge		TFS Eindhoven	MPIB Martinsried	MPIB Martinsried
Electron Gun	XFEG		XFEG		CFEG	XFEG	XFEG
Camera	Gatan K3		Gatan K3		Falcon 4	Falcon 4	Falcon 4
Slit Width (eV)	20		20		10	10	10
Voltage (kV)	300		300		300	300	300
Dose (e^-/Å2)	50		50		40	40	40
Defocus range (µm)	-1 to -3		-1 to -3		-1 to -3	-1 to -3	-1 to -3
Pixel size (Å)	1.061		1.061		0.727	0.93	0.93
Movies	5,598		8,339		20,945	41,977	44,606
Initial particles (no.)	908,151		284,123		421,346	514,863	1,298,857
Final particles (no.)	165,679	37,829	38,358	9,929	64,733	50,656	133,989
Symmetry imposed	C3	C1	C3	C1	C3	C3	C3
Map resolution at FSC=0.143 (Å)	2.9	3.5	3.2	4.0	3.3	3.4	2.8
**Refinement**							
Model resolution at FSC=0.5 (Å)	2.7	3.2	3.0	3.5	3.2	3.4	2.9
Model compositions							
Non-hydrogen atoms	25,015	25,316	24,761	24,727	25,020	24,999	25,018
Protein residues	3,060	3,099	3,027	3,024	3,060	3,057	3,060
Ligand (Sugars)	81	80	81	79	81	81	81
Model B factors (Å^2)							
Protein residues	77.5	101.4	85.2	114.3	58.47	35.17	53.37
Ligand (Sugars)	78.5	81.0	79.7	85.1	69.41	53.44	72.55
R.m.s. deviations							
Bond lengths (Å)	0.004	0.004	0.007	0.004	0.006	0.005	0.005
Bond angles (°)	1.124	1.001	1.156	1.003	0.917	0.879	0.918
**Validation**							
MolProbity score	0.77	0.92	0.89	0.98	0.94	0.86	0.93
Clash score	0.34	0.54	0.47	0.59	0.41	0.37	0.63
Rotamer outliers (%)	0.00	0.11	0.00	0.19	0.41	0.15	0.37
Ramachandran plot							
Favored (%)	97.21	96.40	96.57	95.93	95.75	96.54	96.61
Allowed (%)	2.79	3.60	3.43	4.01	4.25	3.46	3.39
Disallowed (%)	0.00	0.00	0.00	0.07	0.00	0.00	0.00
EMRinger score	3.42	2.90	3.18	2.47	3.55	3.40	4.08
