# Supplementary figures and images for: Virion morphology and on-virus spike protein structures of diverse SARS-CoV-2 variants
Source: EMBO J. 2024 Nov 14;43(24):6469–95. doi: 10.1038/s44318-024-00303-1 (PMC11649927; doi:10.1038/s44318-024-00303-1)

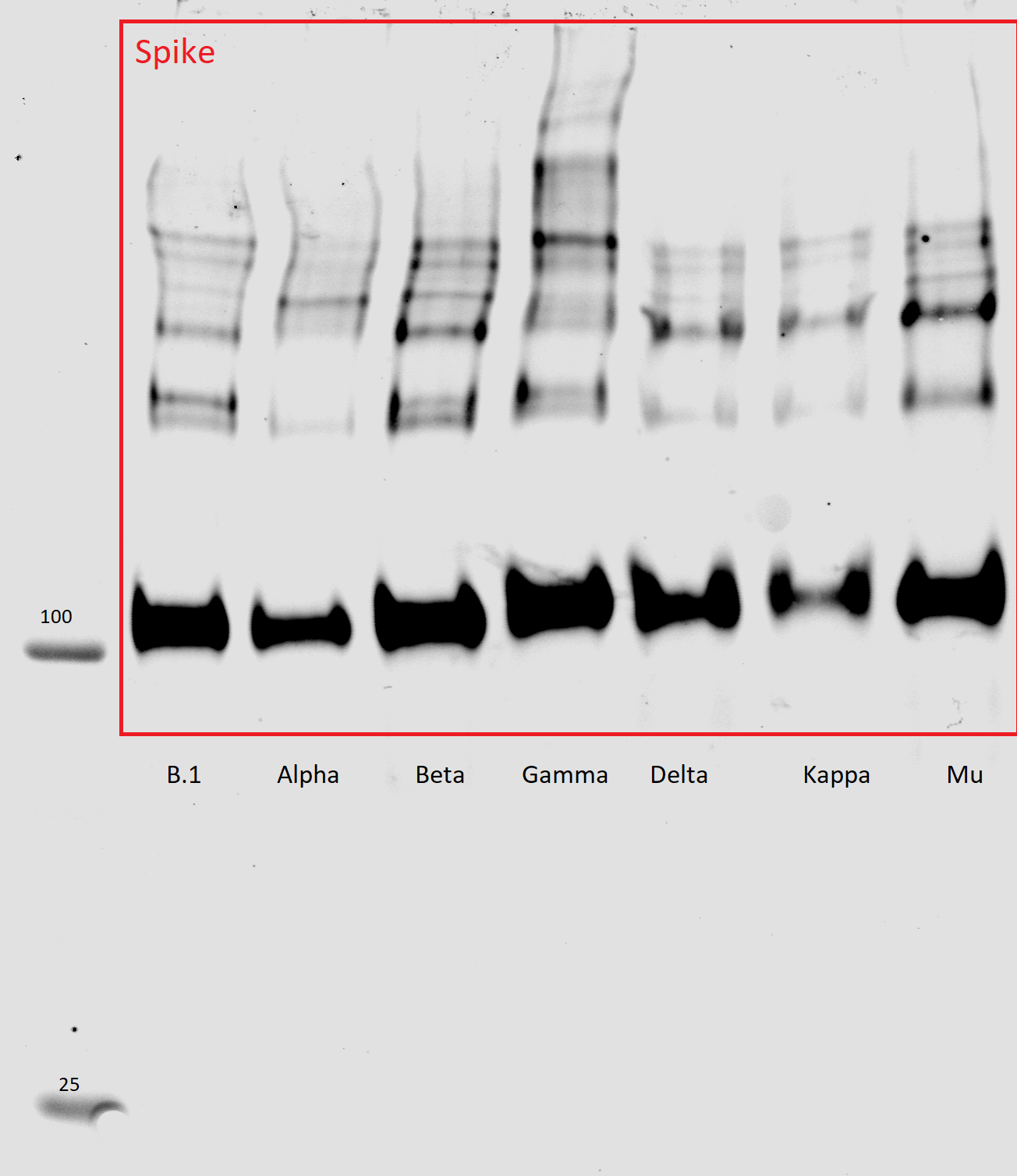

Supplement: Supplementary file 3 — Source data Fig. 1 [file 44318_2024_303_MOESM3_ESM.zip › Figure1_files/Figure 1C 800 channel - Spike.tif]

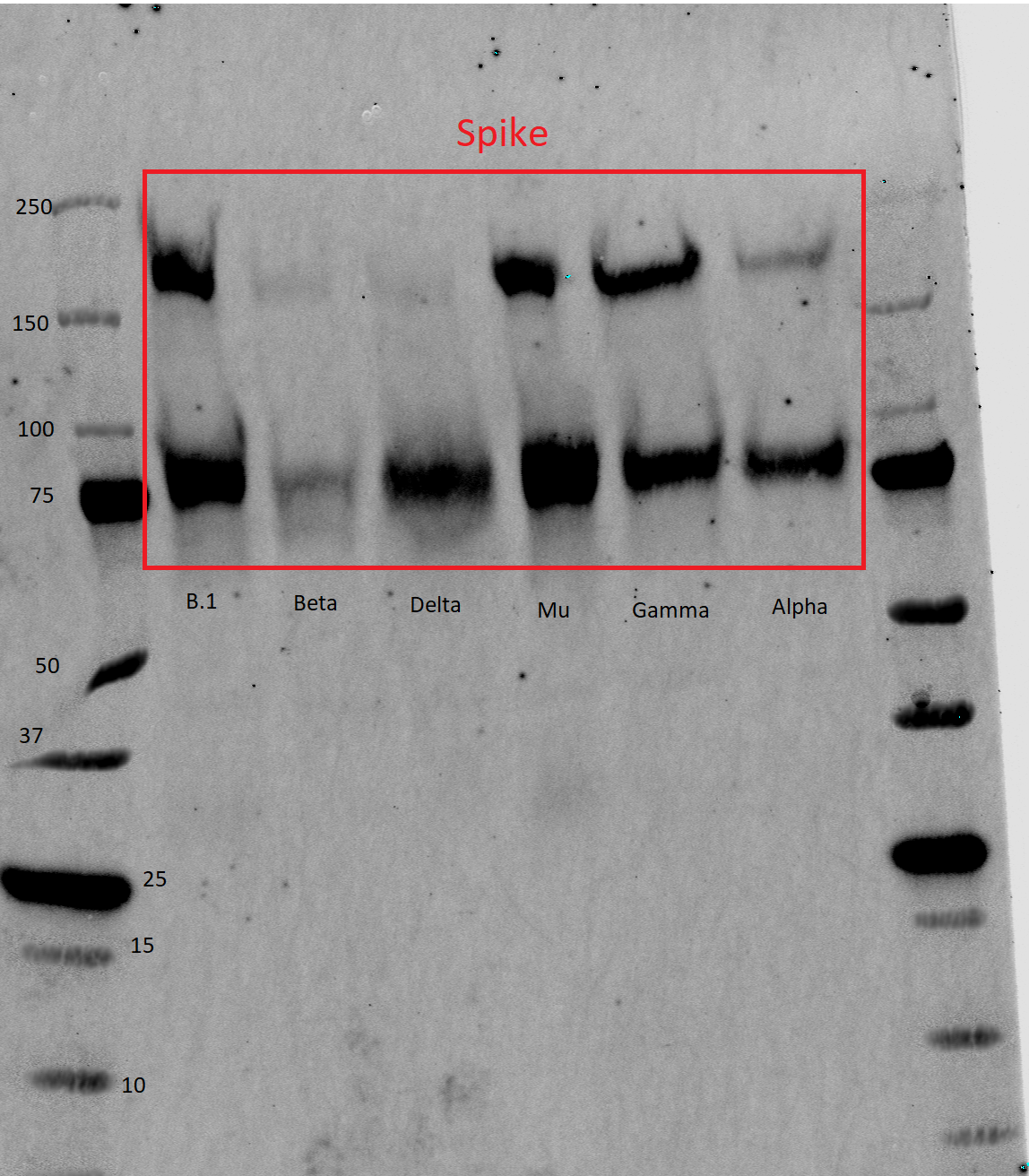

Supplement: Supplementary file 3 — Source data Fig. 1 [file 44318_2024_303_MOESM3_ESM.zip › Figure1_files/Figure 1A Spike.tif]

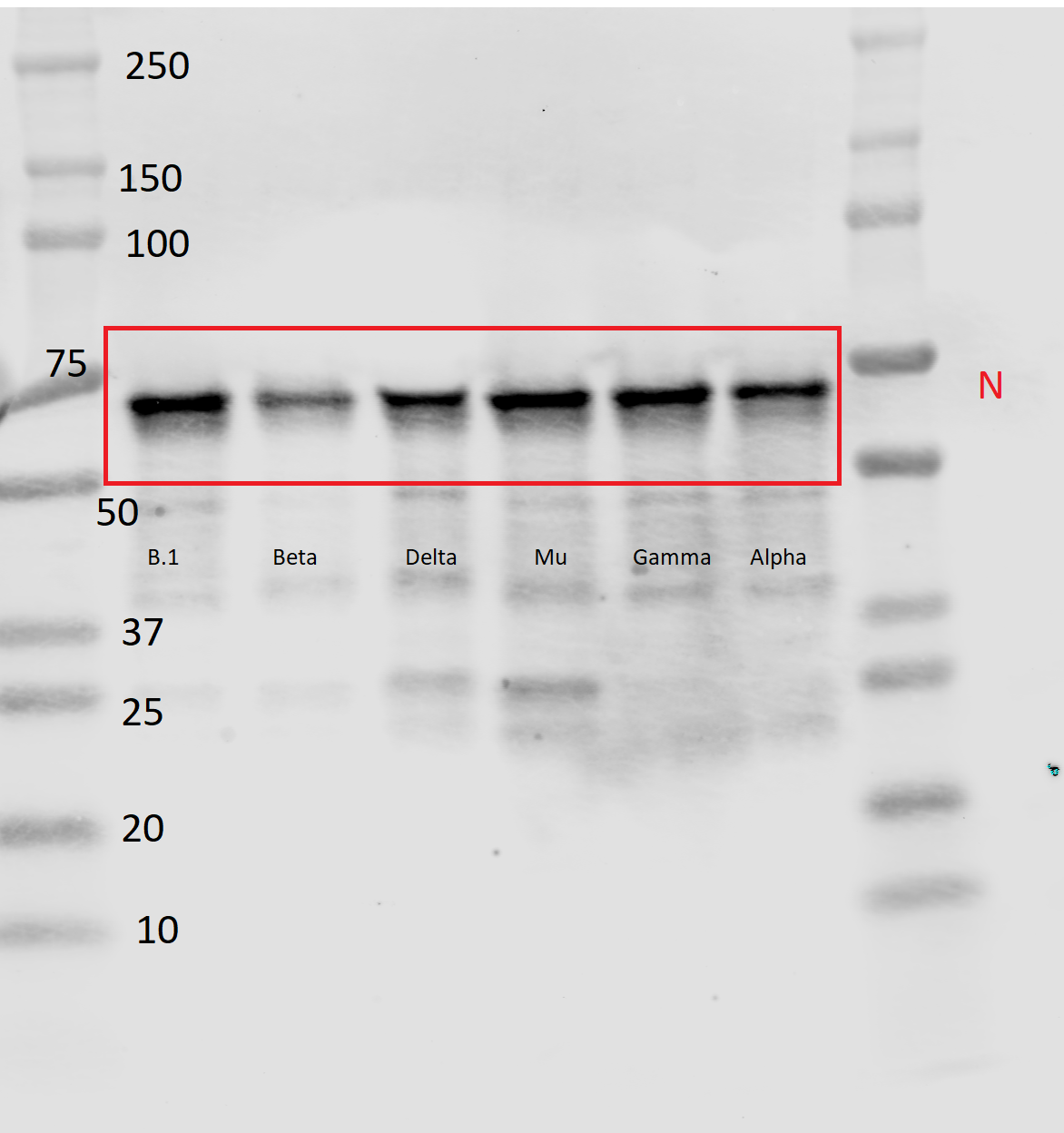

Supplement: Supplementary file 3 — Source data Fig. 1 [file 44318_2024_303_MOESM3_ESM.zip › Figure1_files/Figure 1A N.tif]

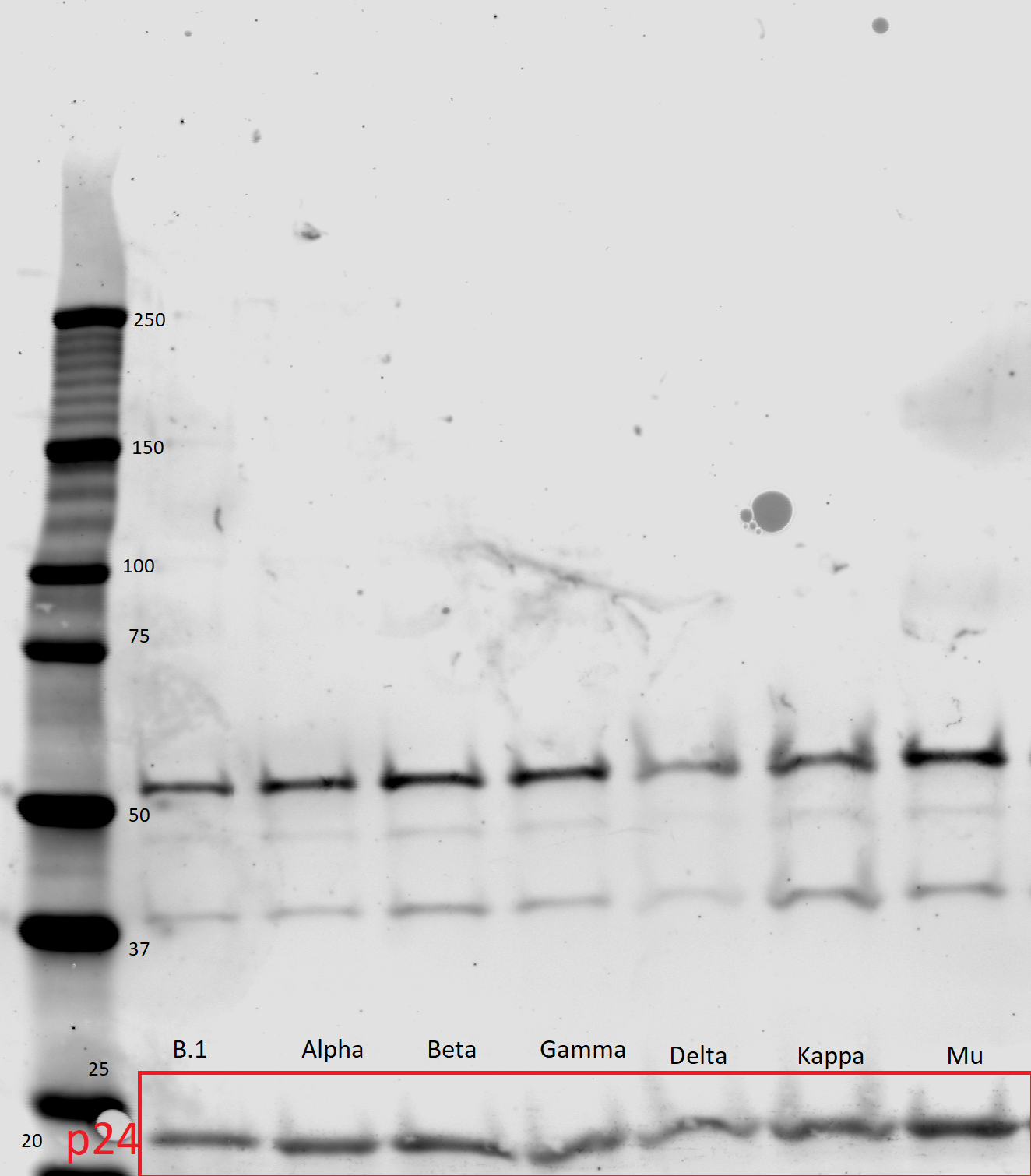

Supplement: Supplementary file 3 — Source data Fig. 1 [file 44318_2024_303_MOESM3_ESM.zip › Figure1_files/Figure 1C 600 channel - p24.tif]

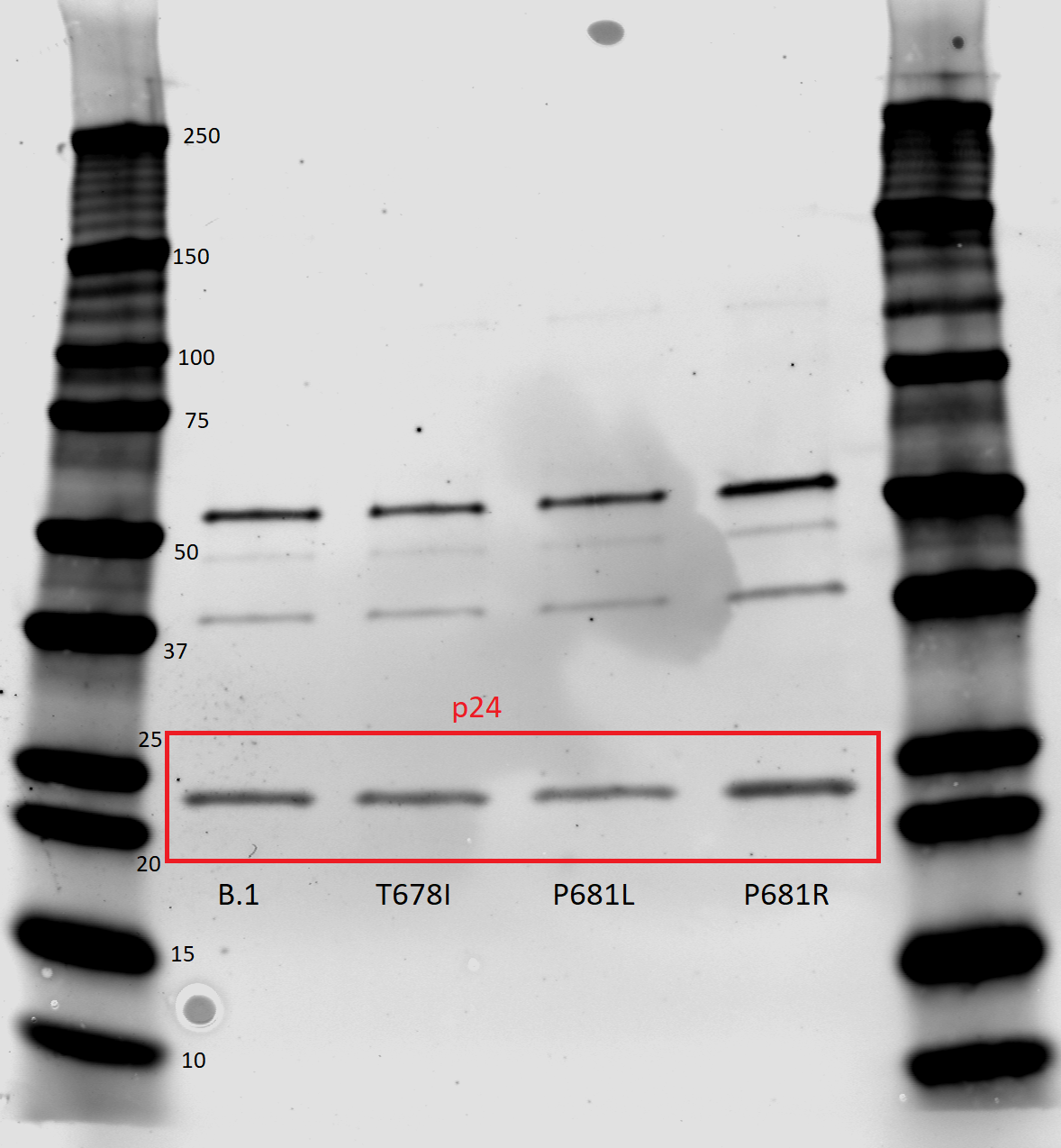

Supplement: Supplementary file 3 — Source data Fig. 1 [file 44318_2024_303_MOESM3_ESM.zip › Figure1_files/Figure 1E 800 channel - p24.tif]

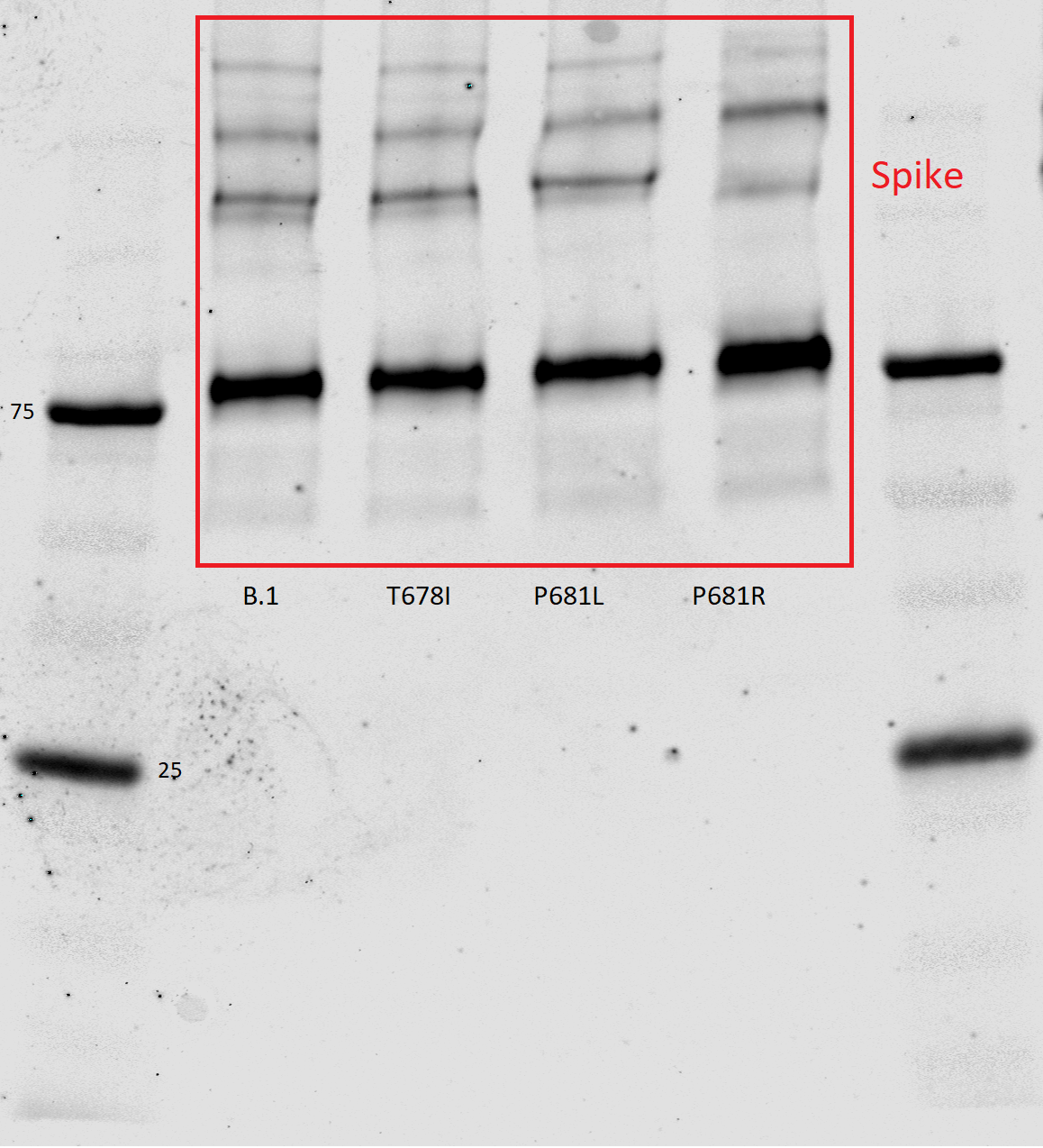

Supplement: Supplementary file 3 — Source data Fig. 1 [file 44318_2024_303_MOESM3_ESM.zip › Figure1_files/Figure 1E 680 channel.tif]

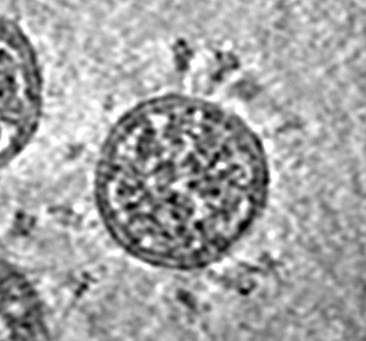

Supplement: Supplementary file 4 — Source data Fig. 2 [file 44318_2024_303_MOESM4_ESM.zip › Figure2_files/Figure_2B_Micr.images/Alpha.tif]

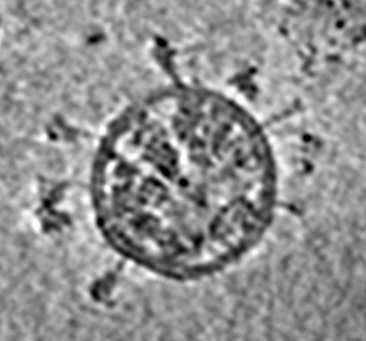

Supplement: Supplementary file 4 — Source data Fig. 2 [file 44318_2024_303_MOESM4_ESM.zip › Figure2_files/Figure_2B_Micr.images/Mu.tif]

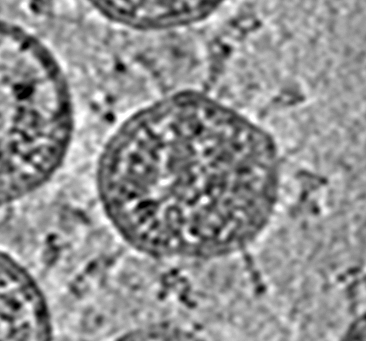

Supplement: Supplementary file 4 — Source data Fig. 2 [file 44318_2024_303_MOESM4_ESM.zip › Figure2_files/Figure_2B_Micr.images/Gamma.tif]

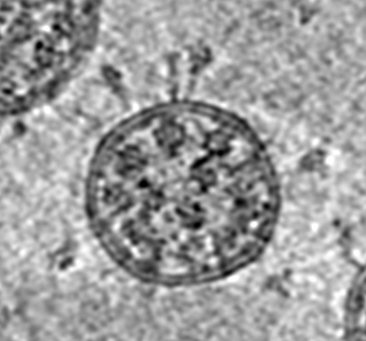

Supplement: Supplementary file 4 — Source data Fig. 2 [file 44318_2024_303_MOESM4_ESM.zip › Figure2_files/Figure_2B_Micr.images/B.1.tif]

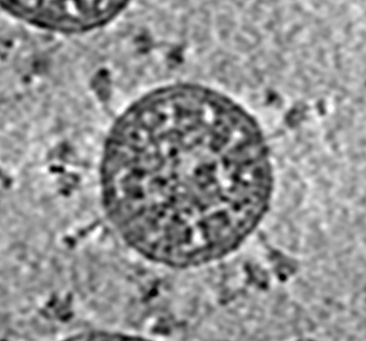

Supplement: Supplementary file 4 — Source data Fig. 2 [file 44318_2024_303_MOESM4_ESM.zip › Figure2_files/Figure_2B_Micr.images/Delta.tif]

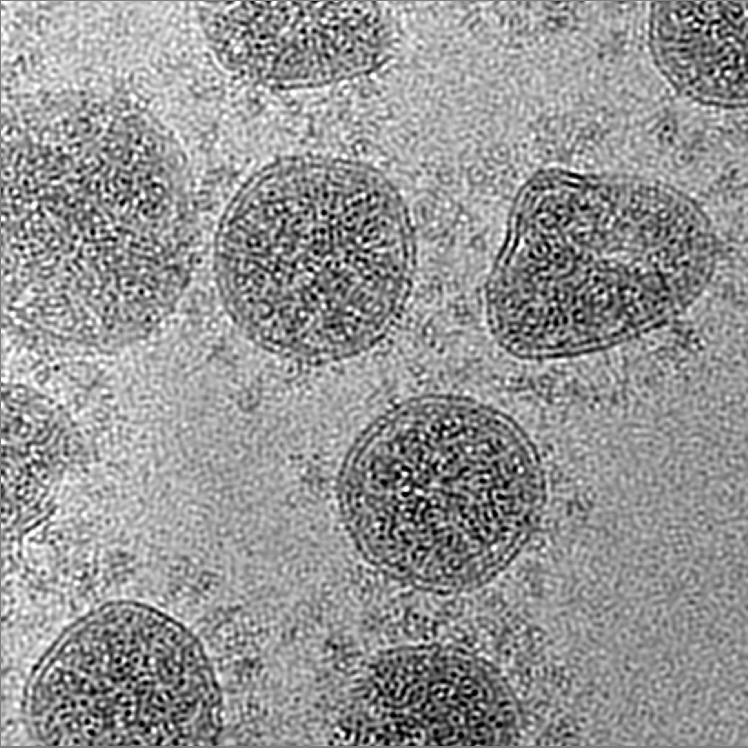

Supplement: Supplementary file 4 — Source data Fig. 2 [file 44318_2024_303_MOESM4_ESM.zip › Figure2_files/Figure_2A_Micr.images/alpha.tif]

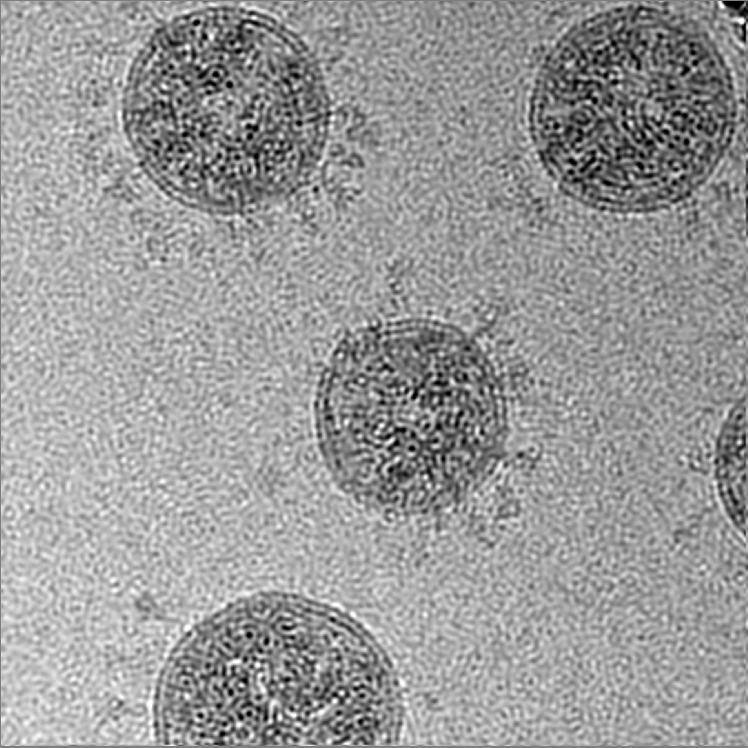

Supplement: Supplementary file 4 — Source data Fig. 2 [file 44318_2024_303_MOESM4_ESM.zip › Figure2_files/Figure_2A_Micr.images/mu.tif]

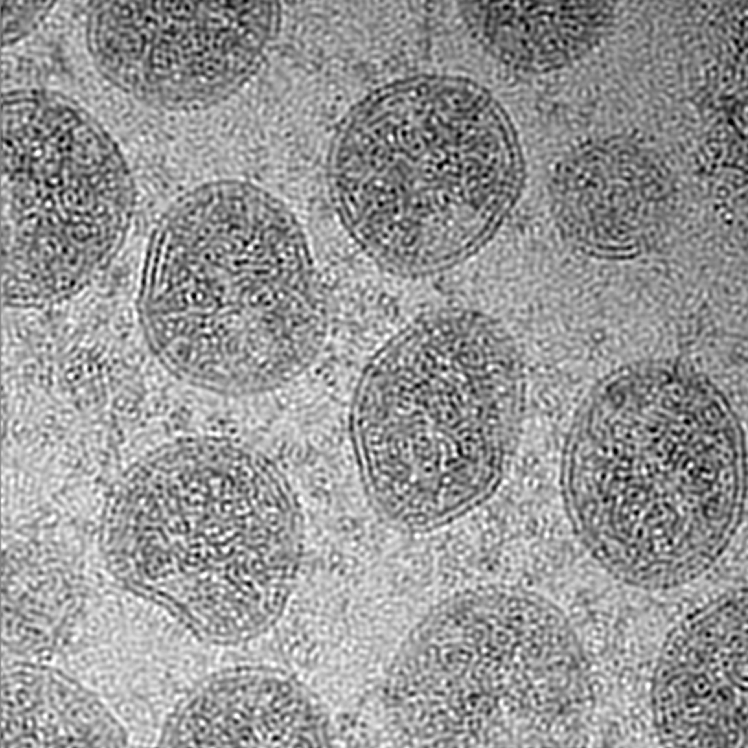

Supplement: Supplementary file 4 — Source data Fig. 2 [file 44318_2024_303_MOESM4_ESM.zip › Figure2_files/Figure_2A_Micr.images/gamma.tif]

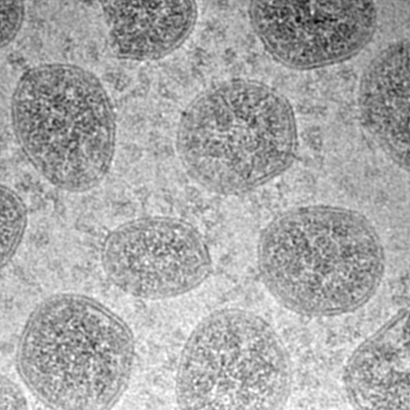

Supplement: Supplementary file 4 — Source data Fig. 2 [file 44318_2024_303_MOESM4_ESM.zip › Figure2_files/Figure_2A_Micr.images/B.1.tif]

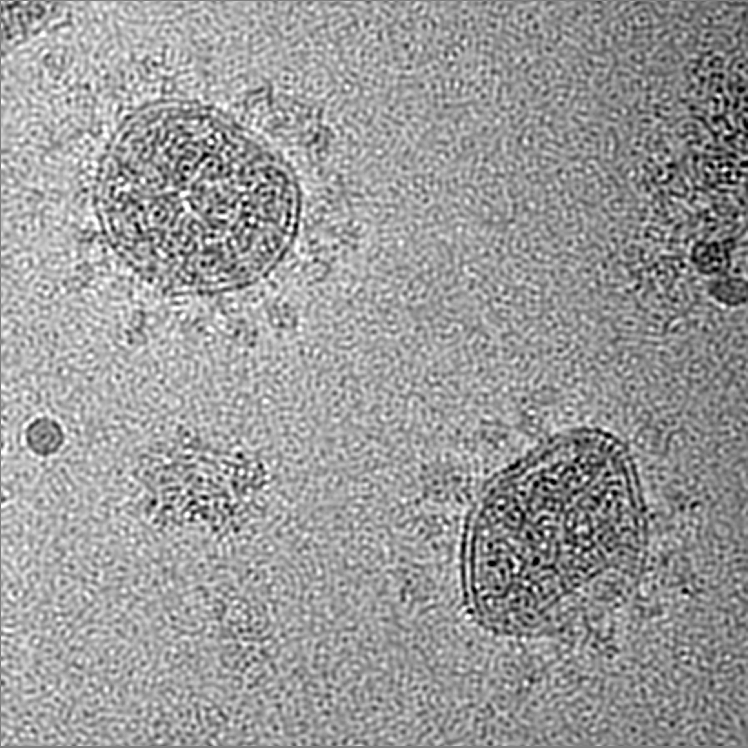

Supplement: Supplementary file 4 — Source data Fig. 2 [file 44318_2024_303_MOESM4_ESM.zip › Figure2_files/Figure_2A_Micr.images/kappa.tif]

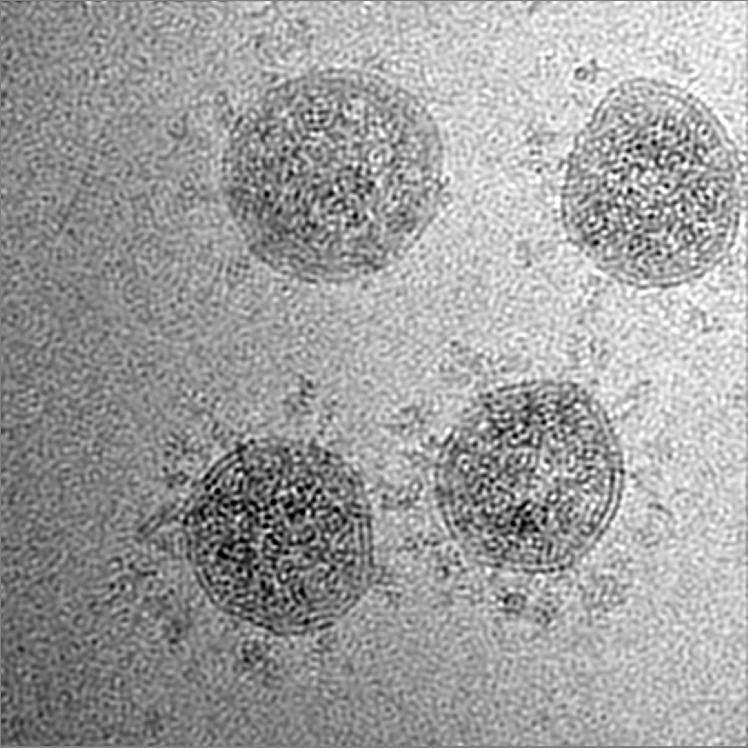

Supplement: Supplementary file 4 — Source data Fig. 2 [file 44318_2024_303_MOESM4_ESM.zip › Figure2_files/Figure_2A_Micr.images/delta.tif]
