# Supplementary material for: Virion morphology and on-virus spike protein structures of diverse SARS-CoV-2 variants
Source: EMBO J. 2024 Nov 14;43(24):6469–95. doi: 10.1038/s44318-024-00303-1 (PMC11649927; doi:10.1038/s44318-024-00303-1)
Supplement: Supplementary file 5 — Expanded View Figures [file 44318_2024_303_MOESM5_ESM.pdf]

Expanded View Figures

Figure EV1. SARS-CoV-2 S mutations and the phylogenetic tree.

(**A**) S structure side and top views. One of the three chains is color-coded according to the color scheme in (**B**) to illustrate the positions of the structural features that are discussed in the manuscript. (**B**) Mutations in S for the variants investigated in this study. Variants are indicated using both WHO labels (Greek letters) and PANGO lineage nomenclatures. Δ indicates amino acid deletions. Secondary structures are color-coded in the index strain (Wuhan-Hu-1) and the residue numbers are marked in the panel. NTD, N-terminal domain; RBD, receptor binding domain; CTD, C-terminal domain; 630 loop, a loop which contains residues 617–644; S1/S2, furin cleavage site; S2′, S2′ cleavage site; FP, fusion peptide; FPPR, furin peptide proximal region; HR1, heptad repeat 1; CH, central helix; CD, connector domain; HR2, heptad repeat 2. (**C**) Neighbor-joining phylogenetic tree of SARS-CoV-2 variants investigated in this study, together with the Omicron BA.1 variant. Scale bar refers to a phylogenetic distance of nucleotide substitutions per site.

© The Author(s)

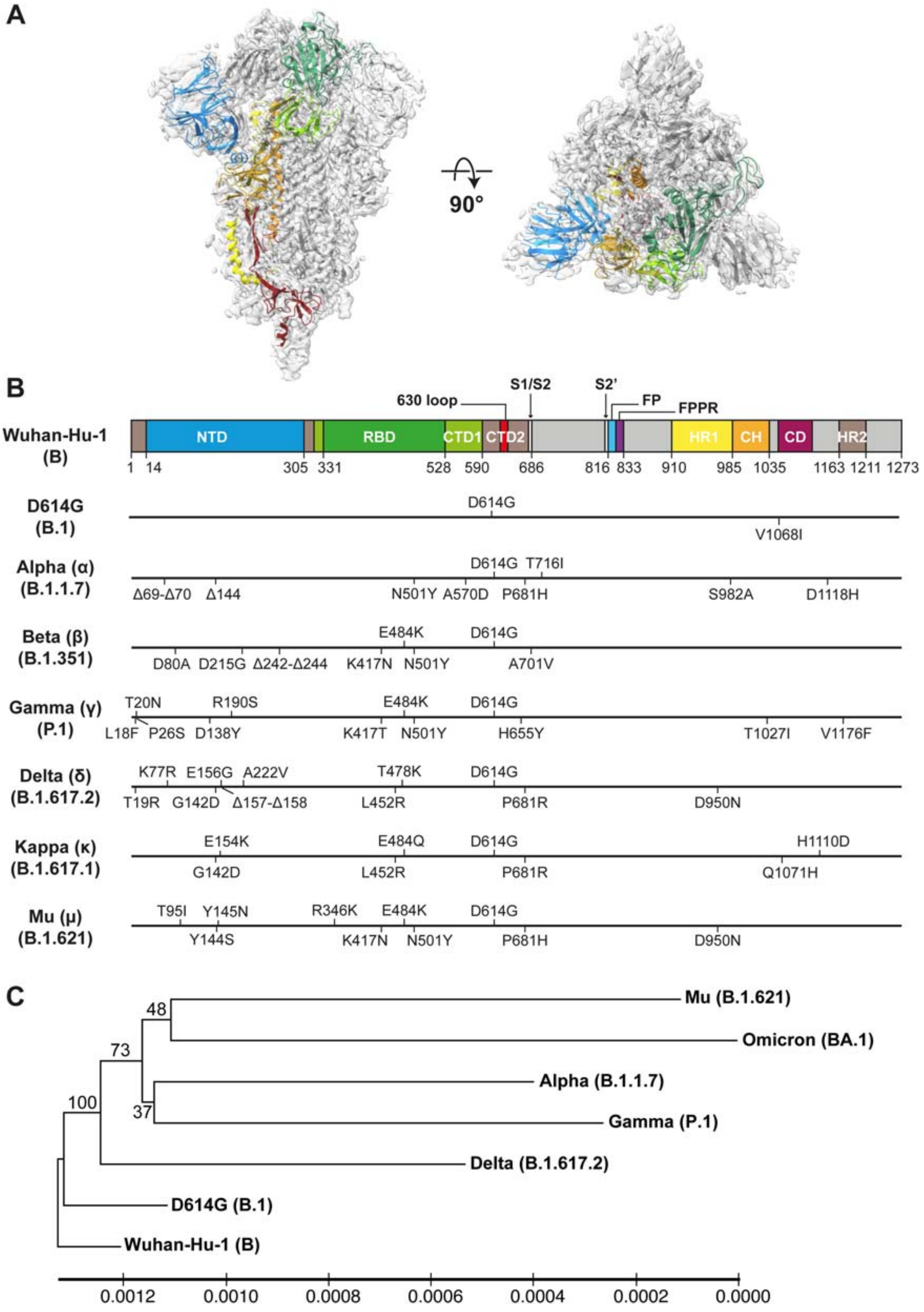
A
90°
B
630 loop
S1/S2
S2'
FP
FPPR
Wuhan-Hu-1
(B)
NTD
RBD
CTD1
CTD2
HR1
CH
CD
HR2
1 14 305 331 528 590 686 816 833 910 985 1035 1163 1211 1273
D614G
(B.1)
D614G
V1068I
Alpha (α)
(B.1.1.7)
D614G T716I
Δ69-Δ70 Δ144 N501Y A570D P681H S982A D1118H
Beta (β)
(B.1.351)
E484K D614G
D80A D215G Δ242-Δ244 K417N N501Y A701V
Gamma (γ)
(P.1)
T20N R190S E484K D614G
L18F P26S D138Y K417T N501Y H655Y T1027I V1176F
Delta (δ)
(B.1.617.2)
K77R E156G A222V T478K D614G
T19R G142D Δ157-Δ158 L452R P681R D950N
Kappa (κ)
(B.1.617.1)
E154K E484Q D614G H1110D
G142D L452R P681R Q1071H
Mu (μ)
(B.1.621)
T95I Y145N R346K E484K D614G
Y144S K417N N501Y P681H D950N
C
48
73
100
37
Mu (B.1.621)
Omicron (BA.1)
Alpha (B.1.1.7)
Gamma (P.1)
Delta (B.1.617.2)
D614G (B.1)
Wuhan-Hu-1 (B)
0.0012 0.0010 0.0008 0.0006 0.0004 0.0002 0.0000

A

	prefusion (n = 15)	postfusion (n = 15)	diameter (nm) (3D, n = 15)	diameter (nm) (2D, n = 100)	total S / surface area (per 1000 nm², n = 15)
B.1	20 ± 10	1.3 ± 1.5	81 ± 7	84 ± 7	1.0 ± 0.5
Alpha (α)	23 ± 12	1.4 ± 1.6	78 ± 7	81 ± 9	1.3 ± 0.6
Gamma (γ)	27 ± 13	1.5 ± 1.8	78 ± 4	81 ± 6	1.5 ± 0.6
Delta (δ)	34 ± 14	2.4 ± 1.9	77 ± 3	81 ± 7	1.9 ± 0.9
Mu (μ)	35 ± 16	2.8 ± 2.2	79 ± 6	80 ± 5	1.9 ± 0.7

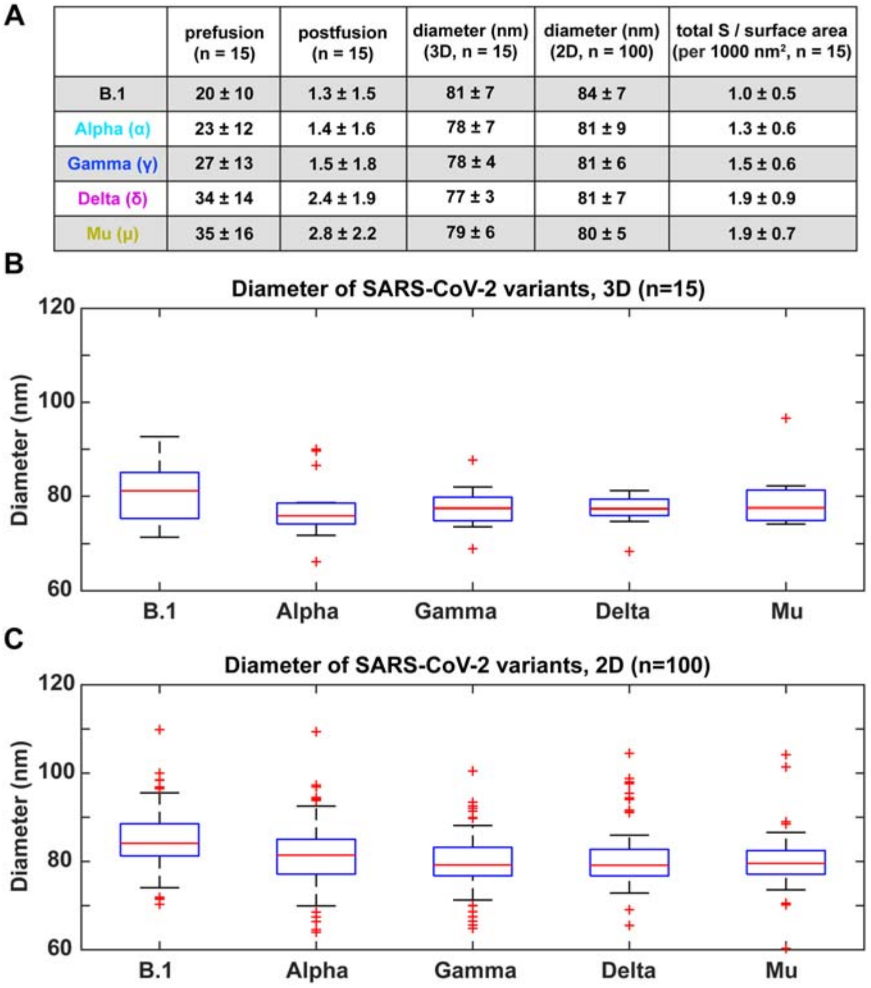

Figure EV2. Virion diameter quantification.

(**A**) The table summarizes the mean numbers of prefusion S, postfusion S, virion diameter (in 3D and 2D measurements), and number of S trimers per unit surface area (per 1000 nm^2). Values are presented as mean ± SD; The number of virions used for quantification is in the column header. (**B**, **C**) Virion diameter measurements from 3D tomographic reconstructions (**B**) and 2D projections (**C**). The n indicates number of virions used for quantification. The box plot represents mean ± SD. A comparative statistical analysis between strains was not performed, because we cannot take possible variation between virus preparations into account. On each box, the central mark (red line) indicates the median, and the bottom and top edges of the box indicate the 25th and 75th percentiles, respectively. The whiskers extend to the most extreme data points not considered outliers. Outliers are plotted individually using the '+' marker symbol.

 © The Author(s)

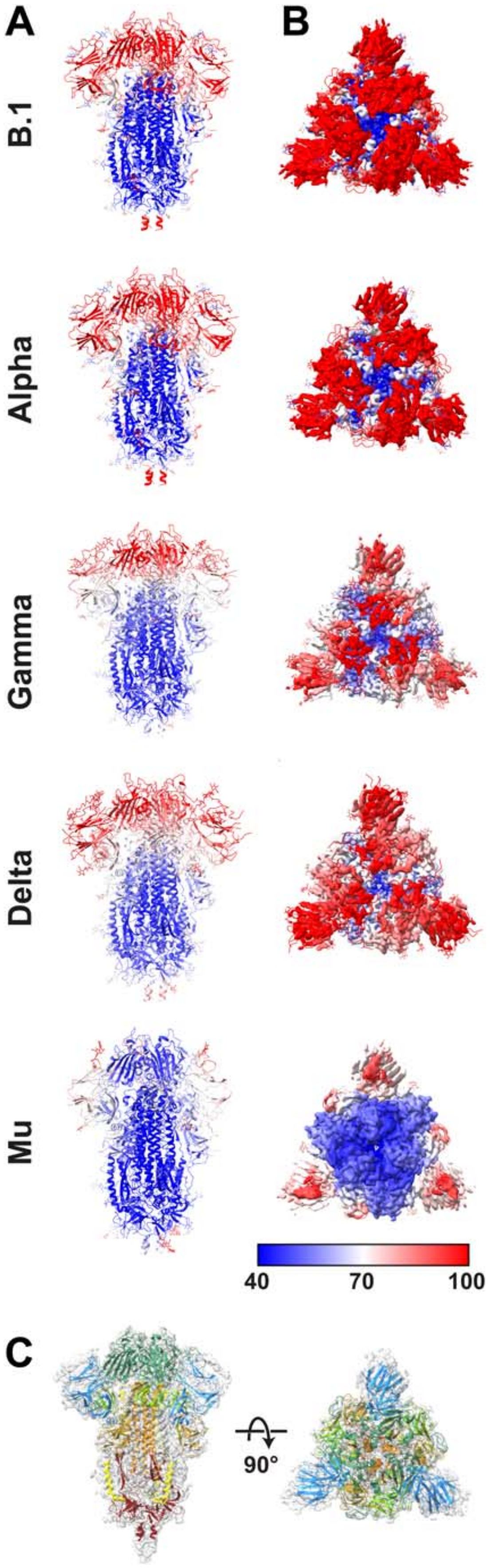

© The Author(s)

Figure EV3. B-factor analysis of S structures.

(**A**, **B**) The modeled structures are color-coded according to the residue specific B-factors from 40 Å^2 (blue) to 100 Å^2 (red). S is shown from the side in cartoon (left column, **A**) and as a top view with the EM map surface colored by b-factor (right column, **B**). The lower the B-factor, the more rigid the protein is. In general, the S2 region is mostly rigid across all the variants, indicated by the low B-factor (blue), while the NTD and RBD are more flexible, indicated by the high B-factor (red). Note, that the Mu variant has a relatively rigid RBD while its NTD remains flexible. (**C**) The secondary structure of S is color-coded according to Fig. EV1.

 © The Author(s)

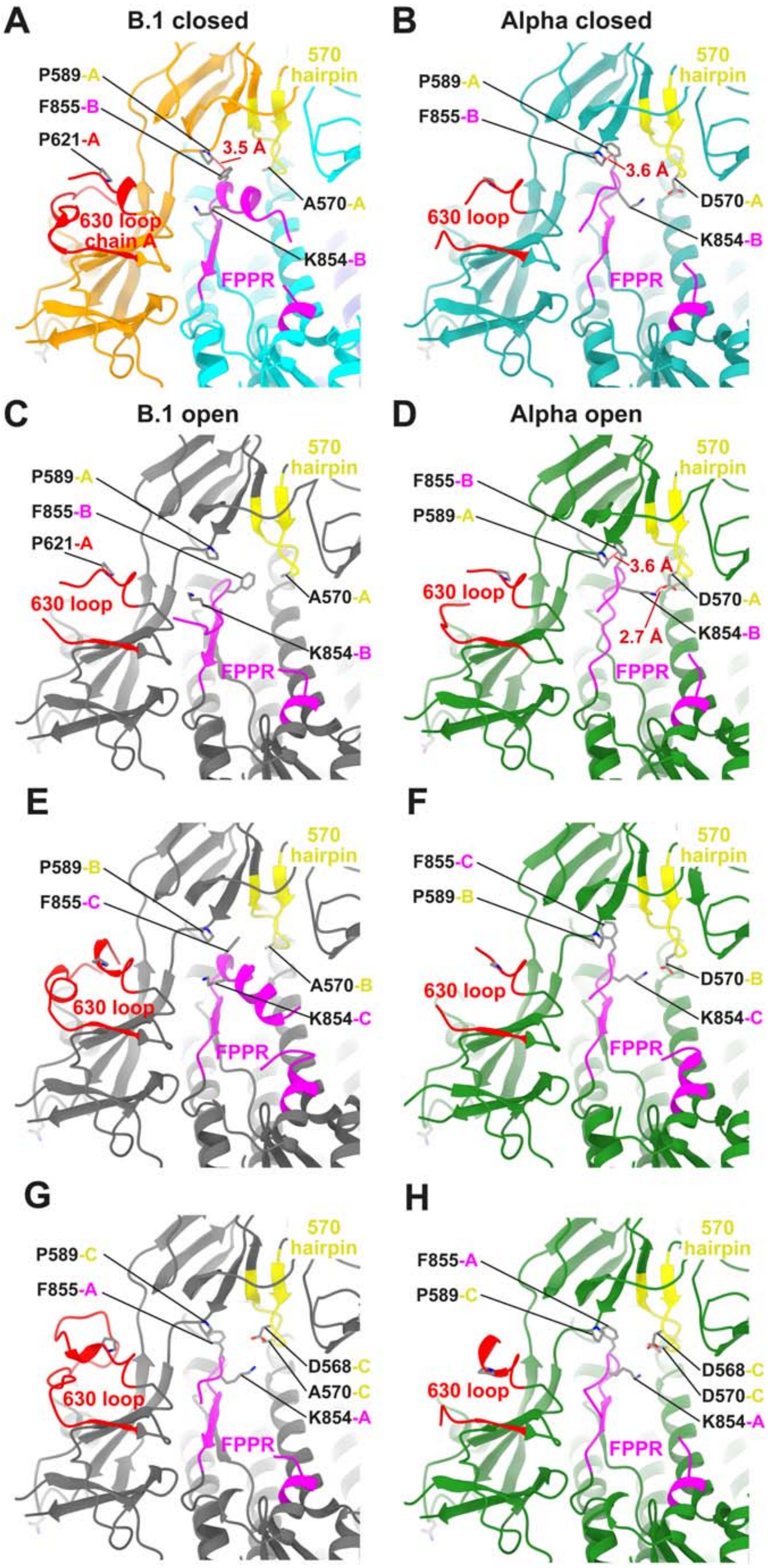
A
B.1 closed
570 hairpin
P589-A
F855-B
P621-A
3.5 Å
A570-A
630 loop chain A
K854-B
FPPR
B
Alpha closed
570 hairpin
P589-A
F855-B
3.6 Å
D570-A
630 loop
K854-B
FPPR
C
B.1 open
570 hairpin
P589-A
F855-B
P621-A
630 loop
A570-A
K854-B
FPPR
D
Alpha open
570 hairpin
F855-B
P589-A
3.6 Å
630 loop
D570-A
2.7 Å
K854-B
FPPR
E
570 hairpin
P589-B
F855-C
A570-B
630 loop
K854-C
FPPR
F
570 hairpin
F855-C
P589-B
D570-B
630 loop
K854-C
FPPR
G
570 hairpin
P589-C
F855-A
630 loop
D568-C
A570-C
K854-A
FPPR
H
570 hairpin
F855-A
P589-C
D568-C
D570-C
630 loop
K854-A
FPPR

Figure EV4. Structural changes modulated by A570D illustrated for all chains.

The three individual chains (ABC) of the 4 structures (B.1 and Alpha, closed and open states) are illustrated here. Color schemes are the same as in Fig. 5. The positions of the three chains are illustrated in (**A**): chain A is in cyan, chain B is in orange, chain C is in blue. (**A–D**) Panels duplicated from Fig. 5 for comparison. (**E–H**) Contacts made by the other chains for B.1 and Alpha open states. The FPPR and interacting residues from each of the three chains from the B.1-open structure are illustrated in (**C**, **E**, **G**); the three chains from the Alpha open structure are illustrated in (**D**, **F**, **H**).

 © The Author(s)

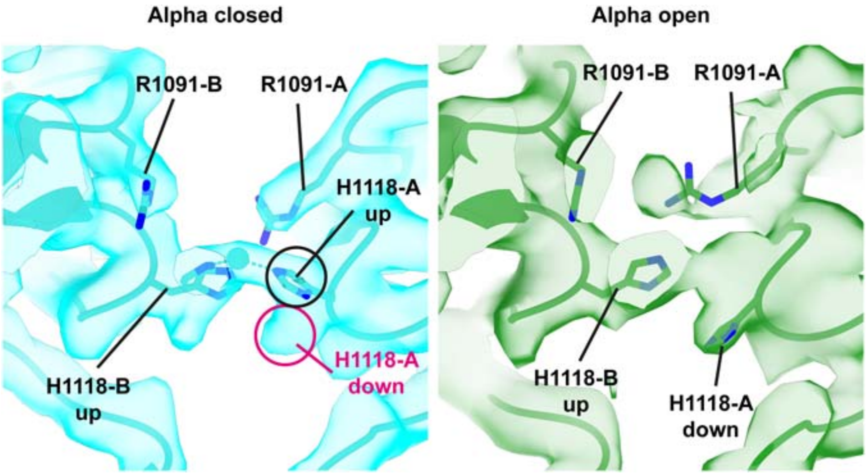

Figure EV5. Structural comparison of S structures in the closed and open conformation at mutation D1118H from Alpha variant.

Left: S structure from Alpha closed state illustrates that H1118 has two conformers, one points upwards (black circle) away from the membrane, and an alternate conformation points downwards (unoccupied magenta circle) towards the membrane. Right: In the open conformation H1118 in chain A (open chain) points primarily downwards and chains B and C points upwards, while R1091 (chain A) has rotated.
